# Functional selectivity and time-dependence of μ-opioid receptor desensitization at nerve terminals in the mouse ventral tegmental area

**DOI:** 10.1111/bph.12605

**Published:** 2014-07-01

**Authors:** J D Lowe, C P Bailey

**Affiliations:** 1 Department of Pharmacy & Pharmacology, University of BathBath, UK; 2 School of Physiology & Pharmacology, University of BristolBristol, UK

**Keywords:** morphine, PKC, GPCR kinase (GRK), opioid, opiate, desensitization, tolerance, nerve terminal, ventral tegmental area (VTA)

## Abstract

**BACKGROUND AND PURPOSE:**

The majority of studies examining desensitization of the μ-opioid receptor (MOR) have examined those located at cell bodies. However, MORs are extensively expressed at nerve terminals throughout the mammalian nervous system. This study is designed to investigate agonist-induced MOR desensitization at nerve terminals in the mouse ventral tegmental area (VTA).

**EXPERIMENTAL APPROACH:**

MOR function was measured in mature mouse brain slices containing the VTA using whole-cell patch-clamp electrophysiology. Presynaptic MOR function was isolated from postsynaptic function and the functional selectivity, time-dependence and mechanisms of agonist-induced MOR desensitization were examined.

**KEY RESULTS:**

MORs located at GABAergic nerve terminals in the VTA were completely resistant to rapid desensitization induced by the high-efficacy agonists DAMGO and Met-enkephalin. MORs located postsynaptically on GABAergic cell bodies readily underwent rapid desensitization in response to DAMGO. However, after prolonged (>7 h) treatment with Met-enkephalin, profound homologous MOR desensitization was observed. Morphine could induce rapid MOR desensitization at nerve terminals when PKC was activated.

**CONCLUSIONS AND IMPLICATIONS:**

Agonist-induced MOR desensitization in GABAergic neurons in the VTA is compartment-selective as well as agonist-selective. When MORs are located at cell bodies, higher-efficacy agonists induce greater levels of rapid desensitization than lower-efficacy agonists. However, the converse is true at nerve terminals where agonists that induce MOR desensitization via PKC are capable of rapid agonist-induced desensitization while higher-efficacy agonists are not. MOR desensitization induced by higher-efficacy agonists at nerve terminals only takes place after prolonged receptor activation.

**LINKED ARTICLES:**

This article is part of a themed section on Opioids: New Pathways to Functional Selectivity. To view the other articles in this section visit http://dx.doi.org/10.1111/bph.2015.172.issue-2

## Introduction

The μ-opioid receptor (MOR), a GPCR (nomenclature follows Alexander *et al*., 2013a), rapidly desensitizes in response to various opioid agonists. The precise molecular mechanism by which this desensitization takes place depends on the opioid agonist used to activate the receptor. For example, high-efficacy agonists such as DAMGO and Met-enkephalin induce MOR desensitization largely through a GPCR kinase (GRK)- and arrestin-dependent mechanism, whereas lower-efficacy agonists such as the prototypical opioid agonist, morphine, induce desensitization largely through a PKC-dependent mechanism (Kovoor *et al*., 1998; Whistler and von Zastrow, 1998; Johnson *et al*., 2006; Feng *et al*., 2011; Grecksh *et al*., 2011; Zheng *et al*., 2011; Bailey *et al*., 2009; Levitt and Williams, 2012; Williams *et al*., 2013).

The vast majority of previous studies examining agonist-induced desensitization of MORs have examined those receptors located on cell bodies of recombinant expression systems (e.g. HEK293 cells) or on cell bodies and dendrites (somatodendritically) of neurons. Yet, in common with most other GPCRs, there is widespread expression of MORs at nerve terminals of neurons in the mammalian CNS. Indeed, generally speaking, if a GPCR is expressed on a neuronal cell body, it is also expressed on the nerve terminal of the same neuron (Miller, 1998).

More recently, there have been reports that MORs located at nerve terminals in the locus coeruleus, periaqueductal grey (PAG) and proopiomelanocortin (POMC) neurons generally do not undergo rapid agonist-induced desensitization (Blanchet and Lüscher, 2002; Fyfe *et al*., 2010; Pennock and Hentges, 2011; Pennock *et al*., 2012).

MORs are expressed both somatodendritically and on nerve terminals of GABAergic neurons in the ventral tegmental area (VTA) (Johnson and North, 1992a; Sesack and Pickel, 1995; Bergevin *et al*., 2002) and activation of MORs in these neurons has been implicated as the primary site of action mediating the euphoric and rewarding properties of opioids (Bozarth and Wise, 1981).

In the present study, agonist-induced desensitization of these receptors has been examined, revealing functional selectivity and time-dependence of MOR desensitization at nerve terminals.

## Methods

### Brain slice preparation

All animal care and experimental procedures were in accordance with the UK Animals (Scientific Procedures) Act 1986, the European Communities Council Directive 1986 (86/609/EEC) and the University of Bath ethical review documents. All studies involving animals are reported in accordance with the ARRIVE guidelines for reporting experiments involving animals (Kilkenny *et al*., 2010; McGrath *et al*., 2010). A total of 97 animals were used in the experiments described here. Male C57Bl/6J mice (3–5 weeks old; bred in-house at the University of Bath, Bath, UK) were decapitated under ketamine- (160 mg kg^−1^) and xylazine- (20 mg kg^−1^) induced anaesthesia. The brains were removed and submerged in ice-cold cutting solution containing (in mmol L^−1^): 20 NaCl, 2.5 KCl, 0.5 CaCl_2_, 7 MgCl_2_, 1.25 NaH_2_PO_4_, 85 sucrose, 25 D-glucose and 60 NaHCO_3_, saturated with 95% O_2_/5% CO_2_. Horizontal brain slices (250 μm thick) containing the VTA were prepared using a DTK-1000 microslicer (Ted Pella, Inc, Redding, CA, USA). Slices were then transferred to artificial cerebrospinal fluid (aCSF) composed of (in mmol L^−1^): 126 NaCl, 2.5 KCl, 1.2 MgCl_2_, 2.4 CaCl_2_, 1.2 NaH_2_PO_4_, 11.1 D-glucose, 21.4 NaHCO_3_ and 0.1 ascorbic acid, saturated with 95% O2/5% CO2 at 32°C, and were left to equilibrate for at least 1 h prior to recording.

## 

### Whole-cell patch-clamp recordings

Slices were submerged in a slice chamber (Warner Instruments, Hamden, CT, USA) mounted on a BX51WI microscope stage (Olympus, Southend-on-Sea, UK) and superfused (2.5–3 mL min^−1^) with aCSF at 32°C. VTA neurons were visualized using oblique illumination. Whole-cell patch-clamp recordings were made using electrodes (3–6 mΩ) filled with (in mmol L^−1^): 85 K-methylsulfonate, 20 KCl, 10 NaCl, 2 MgCl_2_, 10 HEPES, 6 EGTA, 2 MgATP, 0.25 Na_2_GTP and 10 creatine phosphate (pH 7.25, osmolarity 280 mOsm). Recordings of whole-cell currents were filtered at 1 kHz using an Axopatch 200B amplifier (Molecular Devices, Sunnyvale, CA, USA) and were analysed offline using WinEDR and WinWCP (University of Strathclyde, Glasgow, UK) and pClamp (Molecular Devices). Liquid junction potentials of −12 mV were corrected. Cells were excluded from analysis if the series resistance was >25 mΩ or changed more than 25% over the course of the recording.

The VTA contains both dopaminergic and GABAergic interneurons; however, the MORs are located only on the GABAergic cells and the nerve terminals of GABAergic afferent fibres (Johnson and North, 1992a; Sesack and Pickel, 1995). The dopaminergic and GABAergic cells cannot be differentiated visually but may be differentiated through their pharmacological and physiological responses. Dopaminergic neurons have often been characterized by longer action potential durations, a large I_h_ current and a hyperpolarization produced by dopamine (Johnson and North, 1992b; Chieng *et al*., 2011). Action potential duration was measured in current clamp mode and measured as the duration across the base of the action potential. Cells were classified as dopaminergic if the action potential was greater than 1.2 ms (Chieng *et al*., 2011). I_h_ currents were measured in voltage-clamp mode as inward currents in response to a series of hyperpolarizing steps from a holding voltage of −60 to −120 mV in 10 mV increments for 2 s each. Pharmacological responses to dopamine (200 μmol L^−1^) were measured as outward currents from a holding voltage of −60 mV. Cells were classified as dopaminergic if they had an I_h_ current and a dopamine response or an action potential duration greater than 1.2 ms (Johnson and North, 1992b; Cameron *et al*., 1997; Margolis *et al*., 2006; Chieng *et al*., 2011). All of the cells we identified as dopaminergic exhibited a response that was mediated by presynaptic opioid receptors on GABAergic nerve terminals.

In GABAergic cells, the currents carried by the G-protein coupled inwardly rectifying potassium channel (K_IR_3.x, GIRK; nomenclature follows Alexander *et al*., 2013b) were measured in response to brief (60 ms) hyperpolarizing square wave pulses from a holding voltage of −60 to −120 mV administered every 10 s. To enhance the inward current, the extracellular concentration of KCl in the aCSF was increased to 10 mmol L^−1^ and the NaCl concentration decreased accordingly to maintain osmolarity. The current was averaged over 15 ms of the later plateau phase of the response for each trace.

In dopaminergic cells, GABA_A_ receptor currents were measured from a holding potential of −80 mV and pharmacologically isolated with kynurenic acid (2 mmol L^−1^), strychnine (1 μmol L^−1^) and sulpiride (10 μmol L^−1^) and were eliminated in the presence of the GABA_A_ antagonist bicuculline (10 μmol L^−1^; data not shown). Miniature IPSCs (mIPSCs) were recorded in the presence of tetrodotoxin (1 μmol L^−1^) to inhibit action potential driven GABA release. To record evoked IPSCs (eIPSCs), a platinum bipolar stimulating electrode with 0.5 mm spacing (FHC, Inc., Bowdoin ME, USA) was placed locally to the recording site. Slices were stimulated every 10 s at close to the maximal stimulating intensity. To study the effects of only nerve terminal MORs on the evoked response, the contribution of cell body receptors was attenuated by inhibiting GIRK channels with a combination of tertiapinQ (250 nmol L^−1^) and barium chloride (1 mmol L^−1^). This combination of GIRK inhibitors completely blocked dopamine activation of GIRK currents in dopaminergic VTA neurons (data not shown). Although the GIRK inhibitors increased the duration of the eIPSC, they did not affect the opioid-induced inhibition of the eIPSC amplitude. Phorbol 12-myristate 13-acetate (PMA) and dynasore were both bath-applied to slices immediately prior to onset of whole-cell recordings (15–25 min prior to, and during, recordings).

## 

### Data analysis

Electrophysiological data were analysed by paired or unpaired two-tailed Student's *t*-tests or one-way anova with Bonferroni *post hoc* test, as appropriate, using Prism5 (Graphpad Software Inc., San Diego, CA, USA). The Kolmogorov-Smirnov test (KS test) was run online at http://www.physics.csbsju.edu/stats/KS-test.n.plot_form.html. Differences were assumed to be significant at *P* < 0.05.

## 

### Materials

The compounds used in these experiments were supplied as shown below: DAMGO and Met-enkephalin by BaChem (Bubendorf, Switzerland); baclofen, barium chloride, dopamine, dynasore and strychnine by Sigma Aldrich (Poole, Dorset, UK); bicuculline, CTAP, kynurenic acid sodium salt, naloxone, PMA, sulpiride, tertiapinQ and tetrodotoxin citrate by Ascent Scientific/Abcam Biochemicals (Cambridge, UK); CGP54626, GF109203X, and norBNI by Tocris Bioscience (Bristol, UK); morphine by Macfarlan Smith (Edinburgh, UK).

## Results

### DAMGO does not induce rapid MOR desensitization at VTA nerve terminals

**Figure 1:**
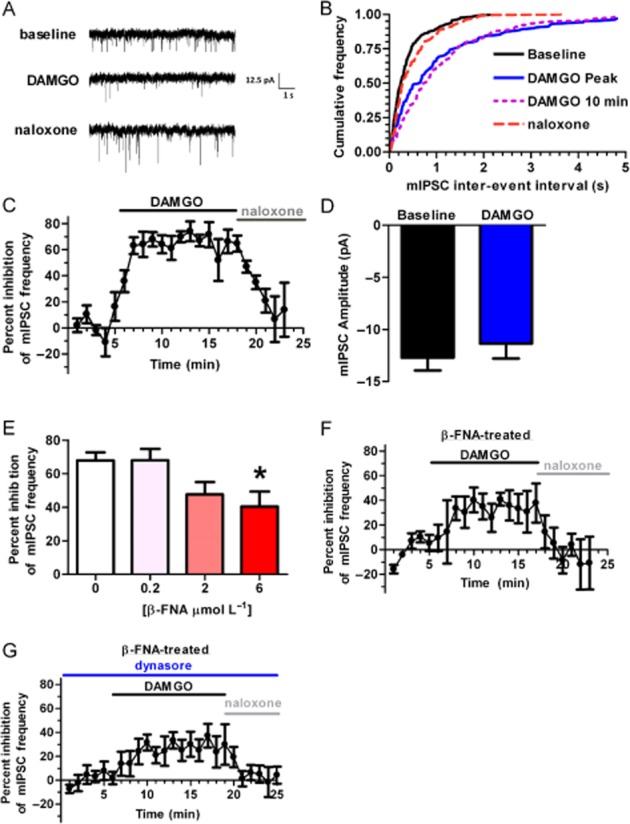
DAMGO does not desensitize nerve terminal MORs. (A) Representative traces of miniature IPSCs (mIPSCs) recorded from a VTA dopaminergic neuron in the presence of kynurenic acid (2 mmol L^−1^), strychnine (1 μmol L^−1^), sulpiride (10 μmol L^−1^) and tetrodotoxin (1 μmol L^−1^). (B) DAMGO (10 μmol L^−1^) application produced a significant decrease in the frequency of mIPSCs measured as an increase in the inter-event interval(s) that was reversed with naloxone (5 μmol L^−1^) (*n* = 3, 53 events per cell, KS test baseline versus DAMGO peak, *P* < 0.001). The peak response of DAMGO was not significantly different from the response after 10 min of continuous agonist exposure. (C) The frequency of mIPSCs was also measured in 1 min bins and normalized to the baseline frequency of mIPSCs. DAMGO (10 μmol L^−1^) produced a reduction in the frequency of mIPSCs that remained stable over the 10-min DAMGO application (*n* = 4). (D) DAMGO (10 μmol L^−1^) did not affect the amplitude of the mIPSCs indicating a presynaptic effect (*n* = 4). (E) The receptor reserve was removed by pretreating slices with the irreversible MOR antagonist β-funaltrexamine (β-FNA) in the presence of the κ-opioid receptor antagonist, nor-BNI, for 30 min. The peak DAMGO-induced inhibition of the frequency of mIPSCs was significantly reduced after 6 μmol L^−1^ β-FNA pretreatment compared with untreated cells (*n* = 4–5, *t*-test, *P* < 0.05). (F) In slices pretreated with 6 μmol L^−1^β-FNA (30 min), the DAMGO (10 μmol L^−1^) response still did not desensitize over a 10-min application (*n* = 5). (G) To prevent rapid receptor recycling which could mask desensitization, slices were pretreated with dynasore (80 μmol L^−1^; >15 min) to inhibit dynamin-dependent receptor internalization. In dynasore and β-FNA pretreated slices, DAMGO (10 μmol L^−1^) inhibition of mIPSC frequency still remained stable over the 10-min application (*n* = 5).

Whole-cell patch-clamp recordings were taken from dopaminergic neurons in the mouse VTA. mIPSCs were recorded, in the presence of tetrodotoxin, in order to isolate any effects of MOR agonists to the nerve terminals of GABAergic afferents (Bergevin *et al*., 2002). Application of the selective MOR agonist DAMGO (10 μmol L^−1^) resulted in a profound decrease in frequency of mIPSCs (Figure 1A–C) with no change in amplitude (Figure 1D) indicative of an action of DAMGO at MORs located on the nerve terminals of GABAergic afferents.

Even when applied at an approximately receptor-saturating concentration (10 μmol L^−1^), DAMGO failed to induce rapid MOR desensitization. After 10 min of continuous bath application of DAMGO, the DAMGO-induced inhibition of mIPSC frequency was the same as at onset of effect (Figure 1C; inhibition at onset of effect: 63 ± 7%, inhibition at 10-min incubation: 66 ± 9%). Cumulative frequency plots (Figure 1B) show a highly significant increase in inter-event interval (i.e. a decrease in event frequency) induced by DAMGO (KS test *P* < 0.001 vs. baseline) that persisted after 10-min incubation and was reversed by the opioid agonist naloxone.

One possible explanation for the apparent failure of DAMGO to induce rapid agonist-induced MOR desensitization is that there is a high receptor reserve at these nerve terminals which occludes any functional loss of response by ongoing MOR desensitization. To investigate this, slices were pre-incubated with the irreversible MOR antagonist β-funaltrexamine (β-FNA) at various concentrations (0.2, 2 and 6 μmol L^−1^) for 30 min before washing and recordings were made. After pre-incubation with 6 μmol L^−1^ β-FNA, the inhibition of mIPSC frequency by an approximately receptor-saturating concentration of DAMGO (10 μmol L^−1^) was significantly reduced (Figure 1E) demonstrating the removal of DAMGO's receptor reserve. DAMGO was then bath-applied for 10 min, following pre-incubation of slices with β-FNA, but no rapid MOR desensitization was observed (Figure 1F; mIPSC inhibition at onset of effect: 34 ± 10%, mIPSC inhibition at 10-min incubation: 38 ± 16%).

As high-efficacy agonists such as DAMGO have been shown to induce profound MOR internalization, recycling and resensitization after agonist-induced desensitization at cell bodies (Arden *et al*., 1995; Law *et al*., 2000; Koch *et al*., 2001); it was possible that extremely rapid and efficient receptor *re*sensitization could mask any apparent DAMGO-induced desensitization. This was studied using dynasore (Macia *et al*., 2006), a cell-permeable inhibitor of dynamin, one of the components of MOR internalization (Patierno *et al*., 2011), which would therefore be expected to inhibit resensitization of any desensitized MORs. However, in the presence of dynasore, there was no time-dependent decline in the DAMGO-induced response (Figure 1G) nor change in the peak response [inhibition of mIPSC frequency 10-min post-DAMGO application: 38 ± 16% (β-FNA alone), 37 ± 10% (β-FNA + dynasore 80 μmol L^−1^)].

### Morphine induces rapid MOR desensitization at VTA nerve terminals when PKC is activated

**Figure 2:**
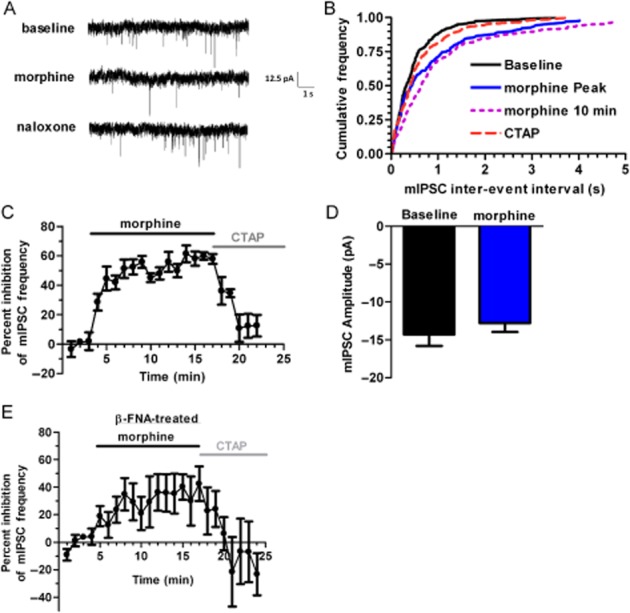
Morphine does not desensitize nerve terminal MORs. (A) Representative traces of mIPSCs recorded from a VTA dopaminergic neuron in the presence of kynurenic acid (2 mmol L^−1^), strychnine (1 μmol L^−1^), sulpiride (10 μmol L^−1^) and tetrodotoxin (1 μmol L^−1^). (B) Morphine (30 μmol L^−1^) application produced a significant decrease in the frequency of mIPSCs measured as an increase in the inter-event interval (s) that was reversed with the MOR selective antagonist CTAP (5 μmol L^−1^) (*n* = 4, 58 events per cell, KS test baseline versus morphine peak: *P* < 0.001). The response to morphine did not desensitize after 10 min of continuous agonist exposure. (C) The frequency of mIPSCs was also measured in 1 min bins and normalized to the baseline frequency of mIPSCs. Morphine (30 μmol L^−1^) produced a reduction in the frequency of mIPSCs that remained stable over the 10-min morphine application (*n* = 5). (D) Morphine (30 μmol L^−1^) did not affect the amplitude of the mIPSCs indicating a presynaptic effect (*n* = 5). (E) In slices where the receptor reserve had been eliminated by pretreatment with β-funaltrexamine (β-FNA; 6 μmol L^−1^, 30 min), morphine (30 μmol L^−1^) inhibition of the frequency of mIPSCs still did not desensitize over the 10-min application (*n* = 10).

As with DAMGO, morphine inhibited the frequency but not the amplitude of mIPSCs (Figure 2A–D), an effect that persisted throughout 10 min of bath application of an approximately receptor-saturating concentration of morphine (30 μmol L^−1^). Figure 2C shows consistent inhibition of mIPSC frequency during bath application of morphine (mIPSC inhibition at onset of effect: 45 ± 8%, mIPSC inhibition at 10-min incubation: 58 ± 5%). These data plotted as a cumulative frequency plot (Figure 2B) show a highly significant increase in mIPSC inter-event interval following addition of 30 μmol L^−1^ morphine (KS test: *P* < 0.001; morphine peak vs. baseline) that persisted during 10-min incubation (‘morphine 10 min’) and was reversed by addition of the selective MOR antagonist CTAP. As before, this apparent lack of desensitization could be because of a high receptor reserve for morphine in this tissue. To investigate this, slices were pre-incubated with β-FNA (6 μmol L^−1^) for 30 min prior to addition of morphine (Figure 2E). Although the time to peak effect was prolonged in slices treated with β-FNA (an effect also seen by Fyfe *et al*., 2010 in PAG neurons, and see Figure 4), no apparent rapid morphine-induced MOR desensitization was seen during the course of a 10-min application.

**Figure 3:**
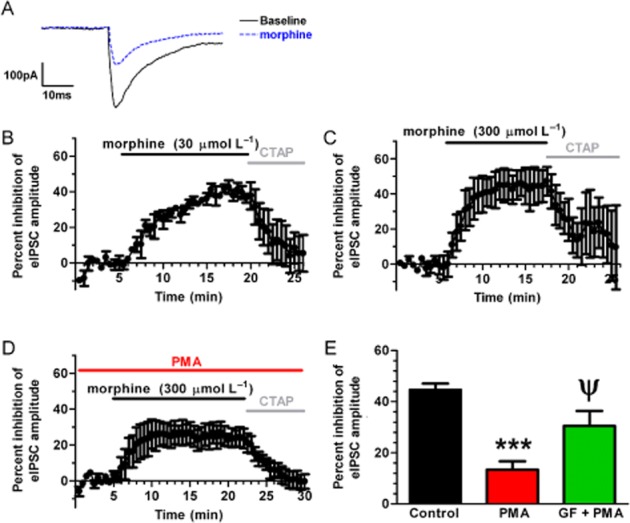
PKC activation enables morphine-induced desensitization of nerve terminal MORs. (A) Representative traces of evoked IPSCs (eIPSCs) recorded from a VTA dopaminergic neuron in the presence of kynurenic acid (2 mmol L^−1^), strychnine (1 μmol L^−1^), sulpiride (10 μmol L^−1^) and the GIRK inhibitors tertiapinQ (250 nmol L^−1^) and barium chloride (1 mmol L^−1^). Application of morphine (30 μmol L^−1^) decreased the amplitude of the eIPSC compared with baseline. (B) Slices were stimulated every 10 s and the amplitude of three consecutive eIPSCs were averaged and normalized to the baseline eIPSC amplitude. In slices where the receptor reserve had been removed with β-funaltrexamine (β-FNA) pretreatment (6 μmol L^−1^, 30 min), morphine (30 μmol L^−1^) produced a reduction in the eIPSC amplitude that was reversed by the MOR selective antagonist CTAP (5 μmol L^−1^) (*n* = 5). However, because the morphine effect was very slow to reach peak, we could not assess acute desensitization over the 10-min application. (C) Increasing the morphine concentration to 300 μmol L^−1^ enhanced the rate of morphine onset in the β-FNA pretreated slices although it still required close to 5 min to reach peak; however, the peak morphine response was not different to the response produced by 30 μmol L^−1^ and was still reversed by CTAP (5 μmol L^−1^). The morphine-induced inhibition of the eIPSC amplitude did not desensitize over the 10-min application (*n* = 5). (D) PKC was activated by treating slices with the phorbol ester PMA (1 μmol L^−1^, >15 min). In β-FNA- and PMA-treated slices, morphine (300 μmol L^−1^) produced less of a reduction in the eIPSC amplitude compared with control β-FNA-treated slices suggesting desensitization had occurred during the relatively slow onset of morphine action. However, once the peak was reached, the response remained stable over the subsequent 10 min of morphine application (*n* = 5). (E) PMA treatment also significantly reduced the peak response to the lower 30 μmol L^−1^ concentration of morphine compared with control β-FNA pretreated slices (*n* = 4–6, anova, *** = *P* < 0.001). The PMA-induced reduction in the morphine response could be reversed with the PKC inhibitor GF 109203X (1 μmol L^−1^) (*n* = 4–6, anova, Ψ = *P* < 0.05 compared with PMA treated).

mIPSCs are caused by spontaneous release of a specific pool of presynaptic vesicles that are released by different mechanisms from those involved in release of neurotransmitter vesicles following an action potential (Ramirez and Kavalali, 2011). It is therefore possible that spontaneous GABA release and activity-dependent GABA release are regulated by different populations of MORs at GABAergic nerve terminals, or by different intracellular signalling processes. We therefore recorded eIPSCs in order to investigate rapid agonist-induced desensitization of MORs at GABAergic nerve terminals that inhibit activity-dependent GABA release from nerve terminals. Figure 3A shows inhibition of the amplitude of eIPSC following bath application of morphine (30 μmol L^−1^). Although it is likely that this effect of morphine to inhibit evoked GABA release is due to activation of MORs located on GABAergic nerve terminals rather than those located somatodendritically, we eliminated the somatodendritic MOR component by performing all eIPSC experiments in the presence of the GIRK blocker tertiapinQ (250 nmol L^−1^) and the potassium channel blocker barium (1 mmol L^−1^) (Johnson and North, 1992a; Jin and Lu, 1999).

**Figure 4:**
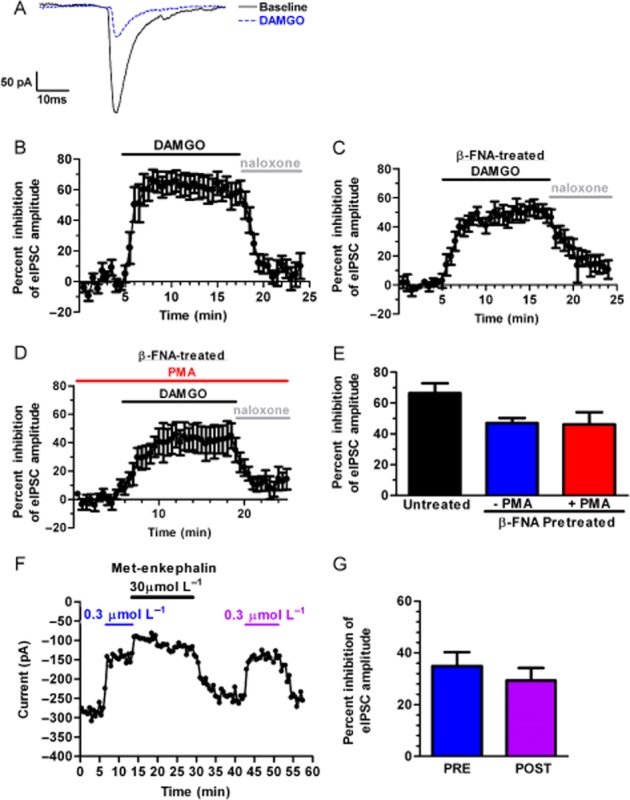
Higher efficacy MOR agonists do not produce rapid MOR desensitization. (A) Representative traces of evoked IPSCs (eIPSCs) recorded from a VTA dopaminergic neuron in the presence of kynurenic acid (2 mmol L^−1^), strychnine (1 μmol L^−1^), sulpiride (10 μmol L^−1^) and the GIRK inhibitors tertiapinQ (250 nmol L^−1^) and barium chloride (1 mmol L^−1^). Application of DAMGO (10 μmol L^−1^) decreased the amplitude of the eIPSC compared with baseline. (B) Slices were stimulated every 10 s and the amplitude of three consecutive eIPSCs were averaged and normalized to the baseline eIPSC amplitude. Application of DAMGO (10 μmol L^−1^) produced a reduction in the eIPSC amplitude that did not desensitize over the 10-min application and was reversed by naloxone (5 μmol L^−1^) (*n* = 8). (C) In slices where the receptor reserve had been removed with β-funaltrexamine (β-FNA) pretreament (6 μmol L^−1^, 30 min), DAMGO (10 μmol L^−1^) inhibition of eIPSC amplitude still remained stable over the 10-min application (*n* = 6). (D) PKC was activated by treating slices with the phorbol ester PMA (1 μmol L^−1^, >15 min). In β-FNA- and PMA-treated slices, the response to DAMGO (10 μmol L^−1^) did not desensitize (*n* = 6). (E) The peak response to DAMGO (10 μmol L^−1^) was significantly inhibited in β-FNA pretreated slices compared with untreated slices (*n* = 6–8, *t*-test, *P* < 0.05) but PMA treatment (1 μmol L^−1^, >15 min) did not affect the peak DAMGO response in β-FNA-treated slices (*n* = 6). (F) eIPSC amplitudes from a representative neuron in an untreated slice where a submaximal concentration of Met-enkephalin (300 nmol L^−1^) was applied before (PRE) and after (POST) a 10-min application of a receptor-saturating concentration of Met-enkephalin (30 μmol L^−1^). (G) The 10-min application of the receptor-saturating concentration of Met-enkephalin (30 μmol L^−1^) did not produce desensitization. The inhibition of the eIPSC amplitude produced by the submaximal concentration of Met-enkephalin (300 nmol L^−1^) was the same before (PRE) and after (POST) the 10-min 30 μmol L^−1^ Met-enkephalin application (*n* = 5).

In the presence of both tertiapinQ (250 nmol L^−1^) and barium (1 mmol L^−1^), and after pretreatment with β-FNA to eliminate the receptor reserve, morphine (30 μmol L^−1^) did not induce any apparent rapid desensitization of nerve terminal MORs, as measured by inhibition of eIPSC amplitude (Figure 3B). However, as with mIPSCs (Figure 2) and as seen by Fyfe *et al*. (2010), the time to peak inhibition induced by bath application of morphine was very slow (approximately 10 min). Even though this concentration of morphine is approximately receptor-saturating (30 μmol L^−1^), this delayed time to peak effect is presumably due to slow access of compound into the synaptic cleft, or slow equilibrium of concentration at the synaptic cleft. It was therefore possible that morphine could be inducing rapid MOR desensitization, and therefore ongoing loss of functional response, that would be masked by the ongoing increase in function caused by slow equilibration of the compound. We therefore repeated the experiment using 300 μmol L^−1^ morphine to reduce time to equilibrium (Figure 3C). Time to peak response was dramatically reduced, with no decline in response over the course of a 10-min drug application. The effects of morphine were reversed by addition of the selective MOR antagonist, CTAP (5 μmol L^−1^), demonstrating that even at this high concentration, the effects of morphine are mediated byactivation of MORs. Moreover, the peak responses induced by 30 μM and 300 μmol L^−1^ morphine were the same (inhibition at 10-min post-application, 30 μmol L^−1^ morphine: 36 ± 3%, 300 μmol L^−1^ morphine: 44 ± 10%).

Previous studies examining desensitization of somatodendritic MORs have shown morphine-induced desensitization to be enhanced when PKC is activated (Bailey *et al*., 2004; 2009; Feng *et al*., 2011; Zheng *et al*., 2011). In the presence of the PKC activator, PMA (1 μmol L^−1^), the peak inhibition of eIPSCs induced by morphine (300 μmol L^−1^) was significantly reduced compared with the absence of PMA (Figure 3D, compare with Figure 3C). Moreover, the peak inhibition of eIPSCs induced by the lower concentration of morphine, 30 μmol L^−1^, was significantly attenuated by the presence of PMA (1 μmol L^−1^), an effect that was inhibited by the PKC inhibitor GF109203X (1 μmol L^−1^) (Figure 3E). Although the inhibition of eIPSCs by morphine in the presence of PMA did not reach a peak response that then declined over time, as is often demonstrated during rapid receptor desensitization, this may have been masked during the prolonged time to peak response (approximately 5 min). Bath application of PMA alone did not induce a significant change in the amplitude of eIPSCs (104.5 ± 1.5% after 10 min PMA vs. baseline, *n* = 5). Furthermore, the reduction in peak effect by PMA was not seen when DAMGO was used as the agonist (Figure 4C–E) demonstrating a form of functional selectivity in the desensitization-enhancing effects of PKC similar to those previously demonstrated at somatodendritic MORs (Kelly *et al*., 2008). Although the effect of PMA was only seen when morphine, not DAMGO, was used to activate MORs, we also investigated whether PMA had a similar effect on presynaptic GABA_B_ receptors and found a slight decrease in the response to a maximal concentration (20 μM) of baclofen (control: 93 ± 2% inhibition, + PMA: 78 ± 5%, *P* < 0.01, *n* = 3–7), but not to a submaximal (2 μM) concentration of baclofen (control: 55 ± 4% inhibition of eIPSCs, + PMA: 51 ± 5%, *n* = 3–7).

### DAMGO does not induce MOR desensitization at VTA nerve terminals but does induce MOR desenstization at GABAergic cell bodies in VTA

The effects of DAMGO on inhibition of eIPSCs were measured. DAMGO (10 μmol L^−1^) induced a rapid inhibition of eIPSC amplitude (Figure 4A,B) that was significantly reduced by pre-incubation of the slices with 6 μmol L^−1^ β-FNA for 30 min (Figure 4C,E; *P* < 0.05 peak DAMGO untreated vs. peak DAMGO β-FNA pretreated). No decline in response was seen over the 10-min duration of application either with or without β-FNA pretreatment (Figure 4B,C). Unlike when morphine was used as the agonist, the peak inhibition caused by DAMGO was completely unaffected by activation of PKC with PMA (Figure 4D,E) demonstrating an agonist-specific effect of PMA.

Although the time to peak onset with DAMGO (approximately 3 min) was much shorter than when morphine was used as the agonist (Figure 3), there still remains the possibility that very rapid MOR desensitization took place during the first 3 min of DAMGO incubation that would be occluded by the time to peak. To overcome this, Met-enkephalin was used; a peptide agonist with similar efficacy to DAMGO (McPherson *et al*., 2010) with the advantage of a more rapid wash-out time in brain slices, compared with DAMGO. A submaximal concentration of 300 nmol L^−1^ Met-enkephalin was applied before and after a 10-min application of a receptor-saturating concentration of Met-enkephalin (30 μmol L^−1^). Thus, if the 10-min application induced MOR desensitization, the response induced by the submaximal concentration would be significantly reduced (Alvarez *et al*., 2002; Fyfe *et al*., 2010). However, the response induced by 300 nmol L^−1^ Met-enkephalin was unchanged by the 10-min application of 30 μmol L^−1^ (Figure 4F,G) further confirming that high-efficacy MOR agonists do not induce rapid receptor desensitization at VTA nerve terminals.

**Figure 5:**
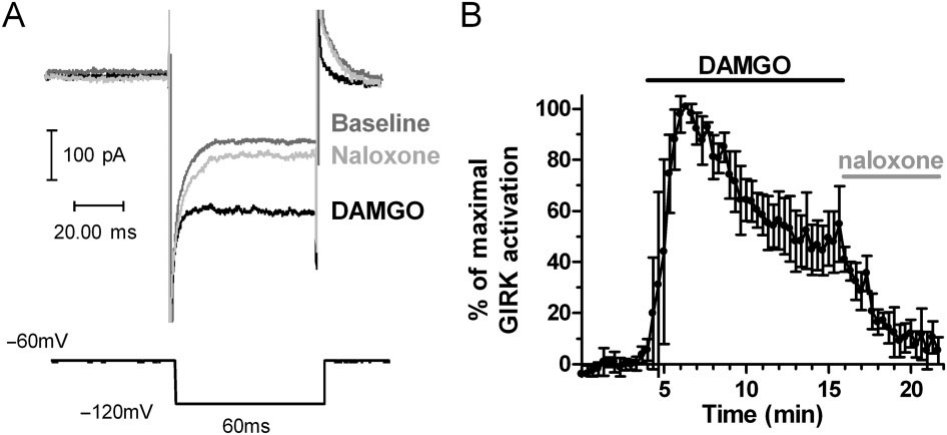
DAMGO produces rapid desensitization of cell body MORs. (A) Representative traces of GIRK currents measured from a VTA GABAergic neuron in response to a hyperpolarizing voltage step compared with baseline in the presence of elevated levels of extracellular potassium (10 mmol L^−1^). DAMGO (10 μmol L^−1^) produced a greater inward current during the voltage step that was inhibited by naloxone (5 μmol L^−1^). (B) Application of DAMGO (10 μmol L^−1^) produced a robust activation of GIRK currents that rapidly desensitized with only 48 ± 9% of the initial current remaining after 10 min of DAMGO application (*n* = 3, anova, *P* < 0.001).

In direct contrast to MORs located at nerve terminals of GABAergic neurons in the VTA, MORs located somatodendritically on GABAergic interneurons undergo robust and rapid agonist-induced desensitization. When whole-cell voltage-clamp recordings were performed from GABAergic interneurons within the VTA, bath application of DAMGO (10 μmol L^−1^)-activated GIRK currents (Figure 5A) rapidly reached a peak that then declined to approximately 50% peak response by 7 min (Figure 5B).

### Long-term incubation with Met-enkephalin does cause desensitization

**Figure 6:**
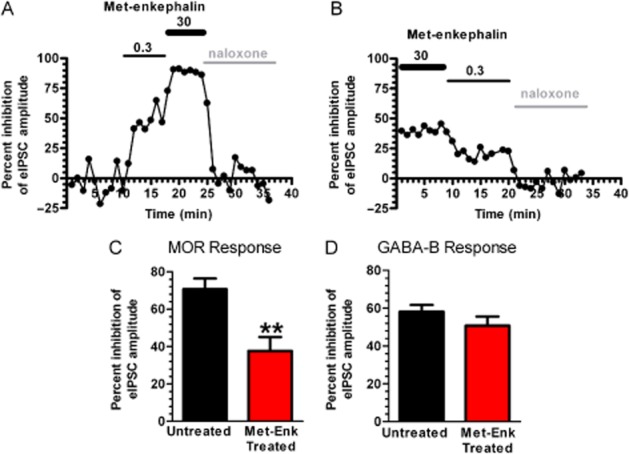
Long-term incubation in Met-enkephalin produces homologous desensitization of nerve terminal MORs. (A) Representative VTA dopaminergic neuron from an untreated slice. Recording taken 7–9 h following incubation in control aCSF. Evoked IPSCs (eIPSCs) were recorded in the presence of kynurenic acid (2 mmol L^−1^), strychnine (1 μmol L^−1^), sulpiride (10 μmol L^−1^) and the GIRK inhibitors tertiapinQ (250 nmol L^−1^) and barium chloride (1 mmol L^−1^). Met-enkephalin application at both submaximal (0.3 μmol L^−1^) and receptor-saturating concentrations (30 μmol L^−1^) decreased the amplitude of the eIPSC which was normalized to the baseline eIPSC amplitude. Effects were reversed by naloxone (5 μmol L^−1^). (B) Representative VTA dopaminergic neuron from a slice that was incubated in Met-enkephalin for 7–9 h starting at 10 μmol L^−1^ and increasing the concentration by 10 μmol L^−1^ every hour to counteract any peptide degradation. Recordings were begun in freshly prepared receptor-saturating concentrations of Met-enkephalin (Met-Enk, 30 μmol L^−1^) which was washed down to a submaximal Met-enkephalin concentration (0.3 μmol L^−1^) and finally the antagonist naloxone (5 μmol L^−1^) was applied. The eIPSC amplitudes were normalized to the eIPSC amplitude in the presence of naloxone. (C) The peak inhibition of eIPSC amplitude produced by the receptor-saturating concentration of Met-enkephalin (30 μmol L^−1^) was significantly reduced in slices that had been pretreated with Met-enkephalin compared with untreated control slices (*n* = 11, *t*-test, *P* < 0.01). (D) The MOR desensitization produced by prolonged Met-enkephalin treatment was homologous. A submaximal concentration of the GABA-B agonist baclofen (2 μmol L^−1^) inhibited the amplitude of eIPSCs to a similar degree as in untreated control slices (*n* = 6–10).

Although neither DAMGO nor Met-enkephalin induce rapid agonist-induced MOR desensitization at GABAergic nerve terminals in the VTA, the ability of prolonged (7–9 h) *in vitro* treatment of Met-enkephalin was studied. Slices were incubated in Met-enkephalin for 7–9 h (10 μmol L^−1^ Met-enkephalin added to the slice incubation chamber every hour to overcome potential peptide degradation, incubation at 32°C). Slices were moved to the recording chamber, superfused with fresh aCSF containing 30 μmol L^−1^ Met-enkephalin, prior to and during the start of each voltage-clamp recording. Upon recording, eIPSC amplitudes were measured in the presence of 30 μmol L^−1^ Met-enkephalin, followed by 300 nmol L^−1^ Met-enkephalin (a submaximal concentration) then naloxone (5 μmol L^−1^). Control slices were incubated at 32°C in drug-free aCSF prior to recording. Figure 6A (control) and Figure 6B (Met-enkephalin-treated) show that 7–9 h of Met-enkephalin treatment results in a profound decrease in the ability of both a submaximal and maximal concentration of Met-enkephalin to inhibit eIPSC amplitude, indicative of desensitization of the MOR taking place during the 7–9 h treatment (pooled data shown in Figure 6C). This desensitization appears to be homologous as the inhibition of eIPSCs induced by activation of presynaptic GABA_B_ receptors by baclofen (2 μmol L^−1^: a submaximal concentration) was not significantly affected by Met-enkephalin pretreatment (Figure 6D) and was not due to naloxone-induced withdrawal effects causing rebound activation of PKA (Bagley *et al*., 2005) as the PKA inhibitor, Rp-cAMPS (100 μmol L^−1^) had identical and non-significant effects in untreated and Met-enkephalin-treated slices (data not shown). Thus, although neither DAMGO nor Met-enkephalin was capable of inducing rapid agonist-induced MOR desensitization (Figures 1,4), profound homologous MOR desensitization occurred with prolonged MOR activation by Met-enkephalin.

## Discussion

The key finding of this study is that the profile of agonist-induced desensitization of MORs located at nerve terminals is profoundly different to those located somatodendritically.

The vast majority of previous papers examining rapid agonist-dependent MOR desensitization have studied those located somatodendritically where high-efficacy agonists induce robust agonist-induced desensitization over the course of several minutes (Christie *et al*., 1987; Kovoor *et al*., 1998; Alvarez *et al*., 2002; Johnson *et al*., 2006; Bailey *et al*., 2009). However, in the present study, the high-efficacy agonists DAMGO and Met-enkephalin failed to induce rapid agonist-induced MOR desensitization at nerve terminals in the VTA. This is consistent with previous studies (Fyfe *et al*., 2010; Pennock *et al*., 2012) in POMC and PAG neurons where short-term treatment with DAMGO or Met-enkephalin similarly failed to induce rapid agonist-induced desensitization. As in those studies, the lack of MOR desensitization was not due to high receptor reserve of nerve terminal MORs as rapid desensitization was still not seen when receptor reserve had been eliminated by pretreatment with the irreversible antagonist, β-FNA. The present data also demonstrated that the lack of rapid presynaptic MOR desensitization is not due to extremely rapid *re*sensitization of previously desensitized receptors (Law *et al*., 2000; Koch *et al*., 2001) as inhibiting dynamin-dependent internalization and recycling was without effect.

However, agonist-induced MOR desensitization was observed with prolonged (7–9 h) activation with Met-enkephalin. The level of desensitization was profound as, even under conditions where the receptor reserve was not removed by pretreatment with an irreversible antagonist, the maximum possible Met-enkephalin effect was reduced by approximately 50%. These data show that nerve terminal MORs are indeed capable of agonist-induced desensitization, but with a far slower time-course compared with those located somatodendritically. In this present study, DAMGO-induced desensitization of MORs located somatodendritically on GABAergic interneurons within the VTA was complete within 7 min. Similarly, DAMGO-induced desensitization of postsynaptic MORs in the rat locus coeruleus had a half-time of 2.3 min (Bailey *et al*., 2003). With the experimental approach taken here, it is not possible to perform a full-time course of the rate and extent of nerve terminal agonist-induced desensitization but studies in neonatal hippocampal neuronal cultures have demonstrated a similar effect with respect to adenosine A_1_ receptors and GABA_B_ receptors (Wetherington and Lambert, 2002a; b). Whereas postsynaptic adenosine A_1_ receptor desensitization was almost complete after 1 h of agonist treatment, presynaptic A_1_ receptor desensitization was not observed until >24 h of agonist treatment and was not complete until ∼72 h; baclofen-induced GABA_B_ desensitization was not observed until >72 h treatment. The reason for why there is an evident time-lag before significant nerve terminal receptor desensitization is observed is not clear. GRK-dependent receptor desensitization is routinely seen as a rapid event (Doll *et al*., 2011), occurring as soon as agonist activation of the receptor occurs, therefore this finding suggests either that ‘slow’ desensitization of nerve terminal MORs takes place via a different, as yet unknown, mechanism to desensitization at cell bodies or that induction or trafficking of GRK proteins or accessory proteins needs to take place before agonist-induced desensitization occurs at nerve terminals.

The fact that there is a time-lag between onset of receptor activation and receptor desensitization with MORs (present study), adenosine A_1_ receptors and GABA_B_ receptors (Wetherington and Lambert, 2002a; b) suggests that this may be a feature common to many G_i/o_-coupled receptors. Although Pennock *et al*. (2012) showed that presynaptic MORs, nociceptin receptors and κ-opioid receptors are all similarly resistant to rapid agonist-induced desensitization in POMC neurons, a subset of presynaptic GABA_B_ receptors did exhibit rapid desensitization.

Studies have shown that MOR desensitization can be homologous or heterologous depending on the cell or treatment type (Harris and Williams, 1991; Blanchet and Lüscher, 2002; Blanchet *et al*., 2003; Llorente *et al*., 2012). In the present study, MOR desensitization at nerve terminals following prolonged Met-enkephalin treatment was largely homologous at least with respect to GABA_B_ receptors, as the response induced by a submaximal concentration of the GABA_B_ agonist baclofen was unaffected by prior Met-enkephalin treatment.

Although rapid agonist-induced desensitization of presynaptic MORs is not induced by the high-efficacy agonists DAMGO and Met-enkephalin, morphine can cause rapid desensitization when PKC is activated. Morphine had a very slow time of onset at nerve terminal MORs, particularly evident when the receptor reserve had been removed, a similar finding to Fyfe *et al*. (2010) in PAG neurons. This is likely to be due to slow time to equilibrium at nerve terminals in the synaptic cleft, and was not seen with DAMGO and Met-enkephalin, even at submaximal concentrations. One potential explanation for this is that morphine is more lipophilic than DAMGO or Met-enkephalin. This slow rate of onset meant that it was possible that morphine-induced desensitization was taking place during the morphine wash-in time. If this were the case, the peak morphine response would be reduced. Indeed, when PKC was activated by the phorbol ester, PMA, there was a significant reduction in peak morphine response. The peak DAMGO response was unaffected by PMA, even under conditions where there was no receptor reserve, meaning that the PMA effect was only observed when morphine was used as the MOR agonist, and therefore likely to be a rapid MOR desensitization effect. This is similar to previous studies examining morphine-induced desensitization of postsynaptic MORs in a variety of cell types where morphine-induced MOR desensitization is largely GRK and arrestin-independent (Kovoor *et al*., 1998; Whistler and von Zastrow, 1998) but can be enhanced with PKC activation (Bailey *et al*., 2004). Although the precise mechanism by which PKC affects morphine-induced MOPr desensitization is presently unclear (Melief *et al*., 2010; Feng *et al*., 2011; Zheng *et al*., 2011; Williams *et al*., 2013), the present study demonstrates that a similar process takes place at nerve terminal MORs in the VTA as takes place at somatodendritic MORs in other cell types. It remains to be seen if this effect is common to all nerve terminals or specific to GABAergic nerve terminals in the VTA.

Although in terms of MOR desensitization the PKC effect was seen when the receptor was activated by morphine, but not by DAMGO, PKC did also cause a slight decrease in the peak response to baclofen, suggesting that other receptors (e.g. GABA_B_) may be similarly affected. One caveat to the PKC finding, however, is that the decrease in morphine response may be due to the effects of PKC to increase probability of transmitter release, meaning that the partial agonist morphine becomes less effective than the full agonist, DAMGO, to inhibit release. Although this is a possibility, we did not see a change in the size of eIPSCs following PMA activation, unlike previous studies that demonstrated increases with the phorbol ester, phorbol dibutyrate, in guinea pig VTA (Bonci and Williams, 1997), although our observed lack of eIPSC amplitude increase could be because we stimulated at near maximal stimulus intensities.

Together, these data show that there are profound differences in MOR desensitization depending on whether the receptors are located somatodendritically or on nerve terminals, both in terms of functional selectivity and time-dependence of effect. Notably, with respect to functional selectivity, it is widely regarded that morphine and other lower-efficacy agonists induce much less rapid MOR desensitization at somatodendritic receptors compared with higher-efficacy agonists. However, the opposite is the case at nerve terminal receptors where, if PKC is activated, morphine can induce significant rapid MOR desensitization whereas DAMGO and Met-enkephalin cannot.

The VTA is the primary site of action of opioids in mediating their euphoric and rewarding effects (Bozarth and Wise, 1981) and so the regulation of MORs in this brain region would be expected to affect tolerance to those properties of opioid drugs. One advantage in examining the difference between presynaptic and postsynaptic MOR desensitization in the VTA is that it is possible to record MOR responses from GABAergic cell bodies as well as from GABAergic nerve terminals. Whereas the postsynaptic MOR responses are necessarily from a single neuron, the presynaptic MOR responses are from numerous GABAergic nerve terminals including GABAergic interneurons as well as afferents from the nucleus accumbens and the rostromedial tegmental nucleus (Jalabert *et al*., 2011; Matsui and Williams, 2011; Xia *et al*., 2011). Nonetheless, the finding that DAMGO caused robust, rapid desensitization of somatodendritic MORs in GABAergic neurons but not of MORs located on GABAergic nerve terminals, coupled with a similar finding in POMC neurons (Pennock *et al*., 2012) suggests compartment-selective regulation of MORs, even within the same neuron, meaning that signalling via presynaptic MORs is much more prolonged than through postsynaptic MORs, at least when high-efficacy agonists are activating the receptor.

*In vivo* studies have demonstrated the functional importance of nerve terminal MORs by inhibiting GIRK signalling, the predominant signalling mechanism of postsynaptic MORs (Vaughan *et al*., 1997). When GIRK signalling was eliminated either with tertiapin or gene-knockout, a significant functional effect of MOR agonists remained (Mitrovic *et al*., 2003; Marker *et al*., 2004; Nakamura *et al*., 2013). Therefore, the processes and mechanisms of MOR desensitization at nerve terminals would be expected to play a critical role in the *in vivo* functional properties of opioid agonists during both short- and long-term agonist treatments. The data reported here suggest that it is not appropriate to extrapolate findings from desensitization of somatodendritic MORs to those located at nerve terminals and that nerve terminal receptors are regulated quite differently.

## Abbreviations

β-FNA: β-funaltrexamine
GIRK: G-protein coupled inwardly rectifying potassium channel
GRK: GPCR kinase
MOR: μ-opioid receptor
PAG: periaqueductal grey
PMA: phorbol 12-myristate 13-acetate
POMC: proopiomelanocortin
VTA: ventral tegmental area
